# Synaptic Reorganization of the Perisomatic Inhibitory Network in Hippocampi of Temporal Lobe Epileptic Patients

**DOI:** 10.1155/2017/7154295

**Published:** 2017-01-02

**Authors:** Lucia Wittner, Zsófia Maglóczky

**Affiliations:** ^1^Institute of Cognitive Neuroscience and Psychology, Research Center of Natural Sciences, Hungarian Academy of Sciences, Budapest, Hungary; ^2^Institute of Experimental Medicine, Hungarian Academy of Sciences, Budapest, Hungary

## Abstract

GABAergic inhibition and particularly perisomatic inhibition play a crucial role in controlling the firing properties of large principal cell populations. Furthermore, GABAergic network is a key element in the therapy attempting to reduce epileptic activity. Here, we present a review showing the synaptic changes of perisomatic inhibitory neuronal subtypes in the hippocampus of temporal lobe epileptic patients, including parvalbumin- (PV-) containing and cannabinoid Type 1 (CB1) receptor-expressing (and mainly cholecystokinin-positive) perisomatic inhibitory cells, known to control hippocampal synchronies. We have examined the synaptic input of principal cells in the dentate gyrus and Cornu Ammonis region in human control and epileptic hippocampi. Perisomatic inhibitory terminals establishing symmetric synapses were found to be sprouted in the dentate gyrus. Preservation of perisomatic input was found in the Cornu Ammonis 1 and Cornu Ammonis 2 regions, as long as pyramidal cells are present. Higher density of CB1-immunostained terminals was found in the epileptic hippocampus of sclerotic patients, especially in the dentate gyrus. We concluded that both types of (PV- and GABAergic CB1-containing) perisomatic inhibitory cells are mainly preserved or showed sprouting in epileptic samples. The enhanced perisomatic inhibitory signaling may increase principal cell synchronization and contribute to generation of epileptic seizures and interictal spikes.

## 1. Introduction

Hippocampal interneurons are classified into different functional groups: dendritic, perisomatic, and interneuron-selective inhibitory cells [1–3]. Dendritic interneurons project their axons to the dendritic region of principal cells and control their input, while perisomatic interneurons innervate the axon initial segment and the somatic region (including proximal dendrites) of principal cells to influence their output. The third group, the interneuron-selective inhibitory cells, innervates exclusively other interneurons [4], regulating the synchrony of hippocampal inhibitory networks [1].

Three main types of perisomatic interneurons can be distinguished in the human hippocampus: parvalbumin- (PV-) positive axo-axonic or chandelier cells, PV-positive basket cells, and Type 1 cannabinoid receptor- (CB1-) positive basket cells also containing cholecystokinin [5]. The two neurochemically different basket cell populations have different role in network oscillations. PV-positive basket cells are known to be specialized to control rhythms whereas CB1-positive interneurons influence the fine tuning of hippocampal synchronies [3]. These two basket cell types are differentiated by their different connectivity, receptors, neuromodulators, and neurotransmitters [6]. PV-containing interneurons are mainly innervated by local principal cells and so are highly efficient in oscillatory functions. In contrast, CB1-positive basket cells receive large amount of subcortical inputs carrying information about the “inner world” of the brain [7] and modulate synchronous ensemble activity accordingly. Therefore, PV-positive basket cells are thought to function as “clockworks” for cortical network oscillations, whereas CB1-expressing interneurons operate as a “plastic fine-tuning device.” Perisomatic inhibition has an important role in the generation and regulation of sharp wave-ripples [8], gamma oscillations [9], and seizure-like activities [10] in rodent hippocampal slice preparations. The first attempts have been made to achieve seizure-control via manipulation of perisomatic inhibition in animal models, by selectively stimulate PV-positive neurons and by transplanting interneuron precursors [11–13]. However, it is still controversial whether perisomatic inhibition of the human hippocampus is increased or decreased during epileptic conditions [11, 14–16]. Here we summarize the fate of PV-immunostained and CB1-immunopositive perisomatic inhibitory interneurons in the hippocampus of temporal lobe epileptic patients. We show that changes in perisomatic inhibition show a complex picture, depending on the hippocampal subregion and the degree of sclerosis.

For these studies we used six control brains from autopsy subjects with no signs of neurological disorders and 57 hippocampi surgically removed from patients with temporal lobe epilepsy. The control brains were removed 2 hours after death; the dissection was performed in the Forensic Pathology Department of the Semmelweis University Medical School. The study was approved by the ethics committee at the Regional and Institutional Committee of Science and Research Ethics of Scientific Council of Health (TUKEB 5-1/1996, further extended in 2005) and performed in accordance with the Declaration of Helsinki. The fixation method and the method of the immunocytochemistry are described in our previous papers [5, 17–20].

## 2. Results

**Figure 1:**
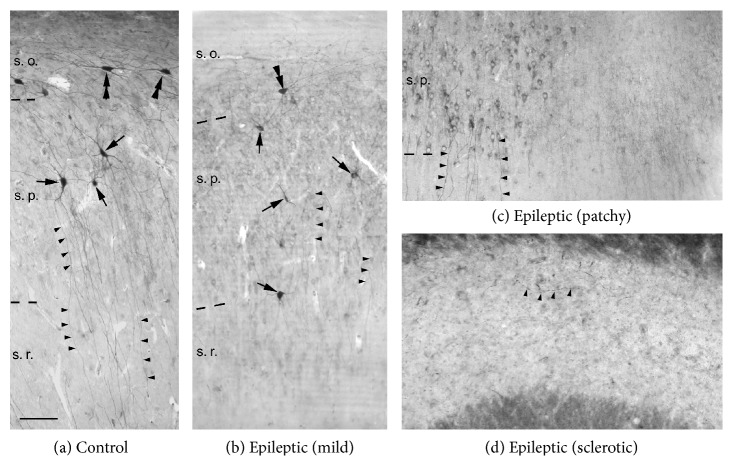
Light micrographs show the distribution of PV-containing interneurons in the human control (a) and epileptic (b–d) CA1 region. (a) Horizontal PV-positive cells (double arrowheads) are present in the stratum oriens (s. o.). Multipolar PV-positive cells (arrows) located in the stratum pyramidale (s. p.) send their dendrites to all layers (arrowheads). (b) In the nonsclerotic epileptic CA1 region (Type 1, mild) the number of PV-positive elements (somata and dendrites) has decreased, mainly visible in the stratum oriens. (c) In the nonsclerotic CA1 region with patchy cell loss (Type 2) the decrease in the number of PV-positive elements is even more pronounced. In several cases, surviving pyramidal cells (left side) accumulate the chromogene diaminobenzidine (giving aspecific staining), possibly due to cellular degeneration processes. (d) In the sclerotic CA1 region (Type 3) only a few PV-stained cells and dendrites are present. Scale bar: 50 *μ*m.

Temporal lobe epileptic (TLE) patients are usually classified based on the degree of hippocampal atrophy. There is still no consensus on the nomenclature of the human hippocampal subregions. The terms hilus, end folium, CA4, and CA3c region are all used and designate slightly different and (in some cases) overlapping areas, all located within the blades of the dentate granule cell layer [17, 21–26]. The transitional area between the CA1 region and the subiculum was also called distal portion of CA1 region in several studies [25, 26] or prosubiculum in others [18, 23, 27]. However, all research groups agreed in establishing two main groups: patients with or without hippocampal sclerosis. Further separation of the epileptic patients was done either based on granule cell excitability, etiology, and surgical outcome [28] or based on complex cell loss pattern [18]. The sclerotic hippocampus was further divided into subgroups, based on the atrophy of the end folium and the CA1 region [26]. In this review, we will follow the groups established in [18] (see Figure 1).

Briefly, (I) Type 1 (mild): it is similar to control, with no considerable principal cell loss in the CA1 region. A slight loss of certain interneuron types is visible in the stratum oriens and hilus. (II) Type 2 (patchy): pyramidal cell loss is in patches in the CA1 region without signs of atrophy. Interneuron loss is more pronounced. (III). Type 3 (sclerotic): the CA1 region is atrophic and shrunken, the principal cell loss is almost complete, and the layers cannot be separated. Interneurons show considerable changes in their distribution and morphology [20].

### 2.1. Changes in the Distribution of Parvalbumin-Containing Interneurons

#### 2.1.1. Control Human Hippocampus

**Figure 2:**
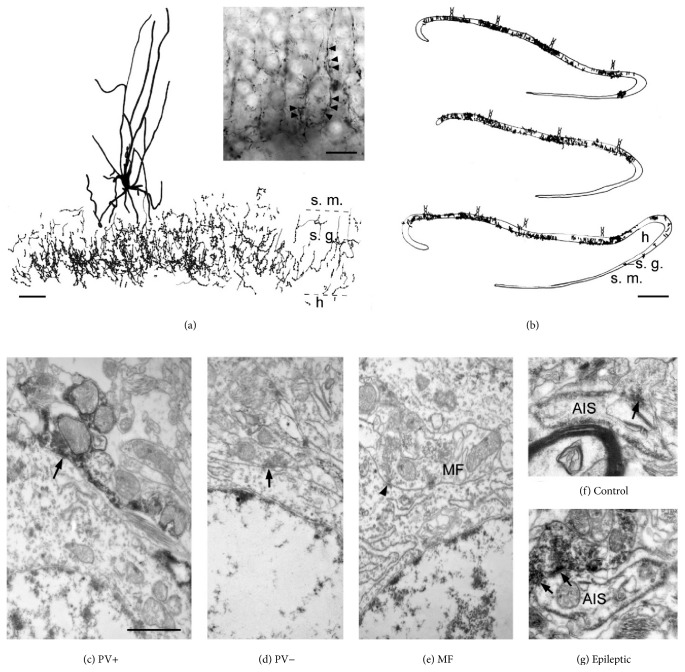
Camera lucida drawings show PV-positive interneuron with its axonal cloud (a) and the inhomogeneous axonal staining (b) in the human sclerotic (Type 3) dentate gyrus. Insert on (a) shows the complex chandelier formations in the dentate gyrus of the sclerotic hippocampus. (b) Dense PV-positive axonal patches are alternated with lack of stained boutons in the granule cell layer of epileptic patients. Schematic cell bodies indicate the location of PV-positive interneuron somata in the stratum moleculare. s. m.: stratum moleculare, s. g.: stratum granulosum, and h: hilus. Perisomatic inhibitory input included PV-positive (c) and PV-negative (d) symmetrical (presumably inhibitory) synapses both in the human control and in epileptic dentate gyrus (arrows). In the sclerotic hippocampus, mossy fibers were also found to form asymmetrical (presumably excitatory) synapses on granule cell somata ((e), arrowhead). Electron micrographs of PV-negative (f) and PV-positive (g) inhibitory synapses terminating on AISs, in the control and epileptic dentate gyrus, respectively. Note the larger bouton in the epileptic tissue, giving a perforated synapse. The somatic and axonal inhibitory synaptic coverage were found to be increased in all epileptic samples. PV+: parvalbumin-positive, PV−: parvalbumin-negative, MF: mossy fiber, and AIS: axon initial segment. Scale bars: (a) 20 *μ*m, insert: 15 *μ*m, (b) 100 *μ*m, and (c–g) 1 *μ*m.

Parvalbumin- (PV-) immunoreactivity was only found in nonprincipal cells throughout the human hippocampus [24, 29, 30]. PV-positive interneurons were located in all layers of the dentate gyrus. They were the most abundant in the hilus and less in the stratum moleculare, and only several cells were visible in the granule cell layer. The majority of the cells were multipolar, but some of them had triangular or fusiform shape. Throughout Ammons's horn PV-positive interneurons were located within or close to the pyramidal cell layer (Figures 1 and 2). They usually displayed large cell body, long smooth dendrites running across all layers. Another characteristic cell type was the fusiform cell with horizontal dendrites found in the stratum oriens (Figure 1(a)). The axons formed a homogenous network in the principal cell layers in both the dentate gyrus and Ammon's horn (Figure 2(a)).

#### 2.1.2. Hippocampi of Temporal Lobe Epileptic Patients

In the epileptic human hippocampus PV-immunoreactivity has decreased to different degree (Figure 1), in correlation with the extent of the severity of the hippocampal sclerosis [17, 18, 24, 25]. The PV-positive neuronal densities determined by the different research groups could be hardly compared because of the differences in the nomenclature of the hippocampus and in the quantification methods. However, the trends were similar: a moderate decrease was found in the number of PV-positive interneurons in the dentate gyrus, the CA1, CA2, and CA3 regions of patients with no sclerosis [17, 25]. The number of PV-positive neurons was the closest to control values (~75% of the control in the dentate gyrus and ~60% in the CA1 region) in the mild group (Type 1) and further decreased (to ~20% of the control in both the dentate gyrus and the CA1 region) in the patchy group (Type 2, Figures 1(b) and 1(c), [18]). In the sclerotic hippocampus (Type 3), the number of PV-positive cells has dramatically decreased, in both the dentate gyrus (to 6% of the control) and the CA1 region (to 21% of control, [17, 18, 25]). Data from Andrioli et al. [25] showed similar trends, although they used different nomenclature to determine the hippocampal subregions. They found significant decrease in the density of PV-positive cells in the nonsclerotic hippocampus only in the polymorphic layer of the dentate gyrus (part of the hilus in our studies) and the CA2 region (both ~50% of control). The density of PV-positive cells was ~65% of the control in the CA3 and ~75% in the CA1 region. The transitional zone between the CA1 region and the subiculum was comprised in the CA1, which we excluded and called prosubiculum. For this reason, they found a reduction of only ~35% in the sclerotic CA1 region, in contrast to our reduction of 79%. They observed PV-positive cell densities ~20% of the control in the polymorphic layer of the dentate gyrus, ~25% in the CA3, and ~50 in the CA2 regions. The cells located in the hilus of the dentate gyrus and the horizontal cells of the stratum oriens in the Cornu Ammonis were the most sensitive to epileptic injury. Their numbers reduced more than that of neurons in other layers [17, 18]. In the mild group, hilar PV-stained cell density was ~50% of the control (which corresponds to the polymorphic layer in [25]), ~13% in the patchy group, and 0.6% in the sclerotic hippocampi. The subiculum is usually considered as a resistant region of the hippocampal formation to epileptic injury. However, the number of PV-containing interneurons has significantly decreased in both the nonsclerotic and sclerotic epileptic samples [25]. Furthermore, the anomalous appearance of the calcium binding protein calbindin (usually present in dendritic inhibitory interneurons, see [1]) has been observed in subicular axo-axonic cell terminals [15, 31]. The existence of a small subgroup of chandelier cells was though demonstrated in the human control and epileptic CA1 region [32].

**Figure 3:**
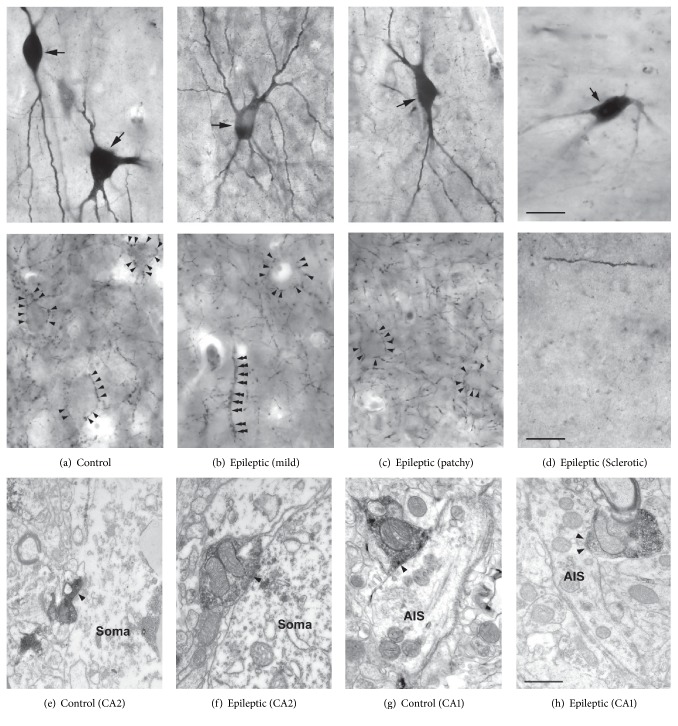
High magnification light micrographs (a–d) show the somata (upper panels) and the axonal cloud (lower panels) of PV-positive cells in the human control (a) and epileptic (b–d) CA1 region. The number of PV-positive elements decreased with the degree of cell loss in the human epileptic CA1 region. PV-stained axons (lower panels) formed a dense network in the stratum pyramidale of the CA1 region as long as their postsynaptic targets, that is, pyramidal cells, are present (b, c). Note the basket-like formation on (a)–(c) (arrowheads) and chandelier-like formation on (b) (double arrowheads). In the sclerotic CA1 region lacking principal cells hardly any PV-positive axons can be seen (d). Scale bar: (a–d) 20 *μ*m. Electron micrographs show PV-stained axonal boutons contacting pyramidal cell somata (e, f) in the control (e) and epileptic (f) CA2 region. Axon initial segments (AISs, (g) and (h)) were the other main targets of PV-positive axons in the human control (g) and epileptic (h) CA1 region. Scale bars: (a)–(d) 20 *μ*m; (e)–(h) 1 *μ*m.

The distribution of PV-stained fibers has also changed in epilepsy (Figures 3(a)–3(d)). In Type 1 nonsclerotic hippocampus the PV-positive axonal network had a similar if not higher density than that of controls [18]. The complexity and the density of chandelier cell axonal formations (Figures 2(a) and 3(a)–3(c)) were also shown to be considerably increased [15]. In all other epileptic cases (patchy and sclerotic groups), the axonal cloud was found to become inhomogeneous (Figures 2(b) and 3). Patches of dense axonal network alternated with areas lacking immunostained elements in the dentate granule cell layer (Figure 2(b), [15, 17–19]). Despite the general reduction of PV-immunostaining, some PV-positive basket and chandelier formations remained both in the dentate gyrus and the Cornu Ammonis regions (Figure 3), which were considerably more complex, than in the control [15]. PV-stained fibers could be hardly seen within the regions with severe principal cell loss (the sclerotic CA1 region and the CA3c/hilar region; see Figure 3(d)), although the presence of very few PV-positive cell bodies and dendrites was shown [15, 18]. In the transition zone between the sclerotic CA1 and subiculum (also called prosubiculum) hypertrophic, very complex PV-stained basket formations were observed [31].

*Electron Microscopy of PV-Containing Synapses*

*(1) Control*. PV-positive somata showed the characteristic features of interneurons at electron microscopic level [30]. They displayed nuclear infolding, intranuclear rods and sheets, large cytoplasm containing numerous organelles, and lipofuscin granules. They received both symmetrical (inhibitory) and asymmetrical (excitatory) axosomatic synapses. The dendrites were usually smooth and received large numbers of asymmetrical synapses [30].

PV-positive axon terminals formed symmetrical synapses with mainly principal cell somata, proximal dendrites, and axon initial segments (AISs, Figures 3(e)–3(h)). The distribution of the target elements of PV-axon terminals showed some differences between the dentate gyrus [17] and the CA1 region [18] of the human epileptic hippocampus. Granule cell somata were more frequently innervated by PV-positive axon terminals than CA1 pyramidal cell bodies, whereas the proportion of PV-stained boutons innervating AISs of granule and CA1 pyramidal cells was similar (Table 1). Lower ratio of PV-immunopositive terminals contacted dendrites and spines in the dentate gyrus than in the CA1 region (Table 1).

*(2) Epileptic*. PV-positive cell bodies and dendrites were examined at electron microscopic level in the epileptic dentate gyrus and CA1 region. The subcellular characteristics of PV-stained cells remained unchanged in the epileptic tissue. The input characteristics, that is, the large amount of asymmetrical synaptic input, were found to be similar to control. However, sprouted mossy terminals were found to terminate on PV-positive cells in the stratum moleculare of the dentate gyrus. Furthermore, PV-positive dendrites were partly covered by glial elements in the sclerotic CA1 region [18].

The target selection of PV-positive axons has been slightly modified in epilepsy. Dentate granule cell somata were contacted in a lower AIS contacted by a higher ratio than in the control (Table 1, [17]). The target distribution did not change systematically in the epileptic CA1 region. High variability was found between the different subjects of all groups (control and epileptic with mild and patchy cell loss, [18]).

*(3) Somatic Input of Hippocampal Principal Cells*. Another approach was used to determine the perisomatic inhibitory innervation of principal cells of the human hippocampus. Measuring the soma/AIS perimeter and the length of active zones of all inhibitory synapses they received helped us to determine the synaptic coverage. This is an estimate of somatic inhibition that does not depend on the PV-content of presynaptic terminals (for the exact method see [17, 18]) and measures all inhibitory synaptic inputs coming from both (PV+ and CB1+) basket cell populations. Briefly, we analyzed about 30 to 50 neighboring principal cell somata or AISs in one electron microscopic section. The perimeter of the somata and AISs, as well as the synaptic length of all boutons contacting them, was measured. The synaptic coverage was provided as *μ*m of synaptic length/100 *μ*m of soma or AIS perimeter. Parvalbumin-immunoreactivity was shown to disappear from surviving inhibitory interneurons in an animal model of epilepsy [33–35]. The inhibitory synaptic coverage includes inhibition coming from perisomatic inhibitory cells as well, which lost their PV-immunoreactivity.

The number of somatic inhibitory synapses contacting dentate granule cells has increased in epilepsy to about 125–135% of control values [17]. The ratio of PV-positive boutons innervating granule cell bodies decreased to about two-thirds in the mild and to about one-third in the patchy and sclerotic epileptic group (Table 2). When measuring the synaptic coverage of granule cell somata, we also received an increase to 126–134% of control value (Table 2). The synaptic coverage of granule cell AISs has increased to a larger extent, than somatic inhibitory input: 215–525% of the control (Table 2, [32]). The ratio of PV-positive boutons contacting AISs has been unchanged in the nonsclerotic groups and was enhanced in the sclerotic hippocampus (Table 2), in accordance with the presence of more complex chandelier formations [15]. We should note that large differences were observed in the ratio of PV-positive boutons contacting AISs between patches containing dense (48.1%) and poor (7.9%) PV-positive fiber network. In the patches lacking PV-containing axonal clouds granule cells were healthy and the synaptic coverage of their AISs was similarly enhanced, than in the patches with strong PV axonal staining.

The somatic input of CA1 pyramidal cells has been investigated only in the control and the nonsclerotic epileptic groups (Table 2, [18]), since pyramidal cells could be hardly found in the sclerotic CA1 region. The somatic synaptic coverage was unchanged in the mild group (Type 1), while the axonal input—in accordance with the presence of very complex chandelier formations [15]—was found to be increased. In the nonsclerotic hippocampus with patchy cell loss (Type 2) both the somatic and axonal inhibitory inputs decreased. We examined patches with and without visible pyramidal cells at electron microscopic level and found that pyramidal cells are present in both regions. They looked healthy in patches containing pyramidal cells, while most of them showed the signs of severe degeneration and cell death in the patches lacking pyramidal cells [18]. PV-positive axonal cloud was present in both types of patches. The ratio of PV-positive boutons was very high in the CA1 region (23 to 45%) compared to the dentate gyrus (11-12%) and increased or remained unchanged in the epileptic samples (39 to 47%). Changes in the synaptic coverage and in the ratio of PV-positive boutons innervating CA1 pyramidal cell AISs were not significant in epileptic tissue [18].

In the dentate gyrus and the CA1 and CA2 regions numerous symmetrical, presumably inhibitory boutons terminated on principal cell bodies. Asymmetrical, presumably excitatory synapses innervating principal cell somata were only seen in the epileptic dentate gyrus with sclerotic hippocampus (Table 2, [17]). Unexpectedly, we also observed frequent contacts on CA2 pyramidal cell bodies made by asymmetrical synapses [19], showing the characteristics of mossy terminals [36]. Therefore, both inhibitory synaptic coverage and excitatory synaptic coverage have been determined for CA2 pyramidal cells (Table 2, Figure 3, [19]). In the epileptic CA2 region the inhibitory synaptic coverage remained unchanged; however, the ratio of PV-positive axon terminals contacting pyramidal cell bodies has dramatically decreased (from 17.5% in control to an average of 2.1% in epileptic tissue). Excitatory synaptic coverage was about the half of inhibitory synaptic coverage in the epileptic cases, with asymmetrical synapses detected on the soma of 50–70% of the examined CA2 pyramidal cells [19].

Inhibitory synaptic coverage of CA2 pyramidal cells exceeded that of CA1 pyramidal cells, which was slightly higher than that of dentate granule cells in the control hippocampus (0.80 ± 0.41 versus 0.64 ± 0.47 versus 0.53 ± 0.64). The inhibitory synaptic coverage of CA1 pyramidal cell AISs was outstandingly high, compared to the somatic and granule cell AIS synaptic coverage (2.34 ± 2.55 versus values of 0.5 to 0.8). The ratio of PV-positive axon terminals innervating the perisomatic region of principal cells was the highest in the CA1 region, compared to other regions of the hippocampus (Table 2, Figures 2 and 3).

In summary, inhibitory input of granule cell somata and AISs was enhanced in the epileptic hippocampus, together with an increase of chandelier formation complexity. Perisomatic inhibitory input is present in the Cornu Ammonis regions as long as pyramidal cells survive. There are though regional differences: the ratio of PV-positive terminals was increased or unchanged in the epileptic CA1 region, while it was dramatically dropped in the CA2 region. We could observe the sprouting of mossy fibers in both the epileptic dentate gyrus and CA2 region. They terminated on the somatic membrane of principal cells in the sclerotic hippocampus, which was never seen in the control tissue [19].

### 2.2. Changes in the Fiber Density of CB1-Immunostained Interneurons

Majority of cannabinoid receptor Type 1- (CB1-) immunostained interneurons were shown to contain the neurochemical marker cholecystokinin (CCK) in the human hippocampus [5, 37]. Most of them are basket cells and terminate on proximal dendrites or cell bodies of principal cells. They can be found in all subfields of the control human hippocampus and dentate gyrus [5]. Unlike PV-positive basket cells, CB1-immunostained cells are present in all fields of the hippocampi of epileptic patients; they can be observed even in the sclerotic CA1 region [38]. This outstanding preservation of CB1-expressing cholecystokinin-immunoreactive cells [20] in epilepsy is comparable to the surviving of calbindin-positive interneurons [32, 39]. CB1 is present on both excitatory and inhibitory terminals [3, 40, 41]. In the present review we have examined only the fate of CB1 receptor-expressing GABAergic cells.

The downregulation of the overall CB1 mRNA (including receptors to be transported to both excitatory and inhibitory synaptic terminals) has been described in the human epileptic hippocampus [38]. Expression of the endocannabinoid molecules (together with CB1 receptor) linked to excitatory network was demonstrated to be decreased, but no reduction of the CB1 receptor associated with inhibitory interneurons could be shown [38]. Different antibodies against CB1 recognize different parts of the receptor and give different immunostaining patterns. One antibody stains all CB1 receptors, present in both excitatory and inhibitory terminals, whereas the other—used in our previous studies—detects CB1 receptor located exclusively on inhibitory terminals [5, 20]. The present review was based on the studies using the latter antibody visualizing CB1 receptors expressed by GABAergic interneurons.

The distribution of synaptic targets of CB1-immunopositive elements was studied in the stratum moleculare of the dentate gyrus in control and sclerotic TLE subjects, where the highest fiber density was present. The postsynaptic targets of CB1-immunopositive terminals were mostly dendrites, cell bodies, and spines, and their ratio was similar in control and epileptic subjects [20]; about two-thirds of the CB1-labeled axons terminated on dendrites, ~14% on cell bodies, and ~13% on spines.

**Figure 4:**
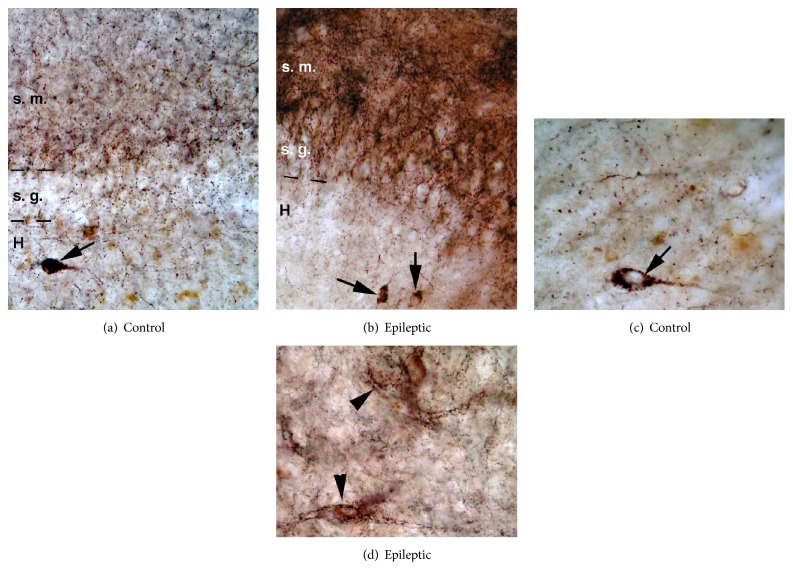
Light micrographs show the distribution of CB1-immunoreactive elements in the human control (a, c) and epileptic (b–d) dentate gyrus. (a) Dense CB1-immunopositive meshwork was present around the dentate granule cells and in the stratum moleculare (s. m.). The hilus (H) contained less CB1-positive terminals. Arrow points to a CB1-positive interneuron. (b) In epileptic patients with hippocampal sclerosis the density of CB1-positive fibers has been increased in the stratum moleculare and granulosum. Note the dispersion of granule cells. Arrows point to CB1-positive interneurons. (c) CB1-immunostained terminals were present in the hilus, in homogenous distribution. Arrow points to a CB1-positive interneuron. (d) In the hilus of the epileptic hippocampus more CB1-positive terminals were present; they often formed dense network around surviving mossy cells or interneurons (arrowheads).

In the nonsclerotic epileptic hippocampi, the distribution of CB1 receptors in the dentate gyrus did not differ significantly from the normal postmortem controls. In contrast, in the dentate gyrus of epileptic patients with sclerotic CA1 region a strong increase in CB1-immunostaining was demonstrated [20]. The density of immunostained fibers increased in the dentate molecular layer (Figure 4) and became inhomogeneous in the hilus forming dense meshwork of terminals around the surviving mossy cells. The density of CB1-positive fibers establishing symmetric synapses was measured by confocal laser scanning microscope, and the elevation of CB1-R-immunostaining was 1,5-fold of the control [20].

The origin of this density increase could be both the elevated number of receptors and sprouting of CB1-R expressing fibers. The sprouting of the axons of a perisomatic inhibitory interneuron type, the PV-containing axo-axonic cells, has been observed in the human epileptic dentate gyrus. However, the increase in the number of receptors in individual terminals cannot be excluded either. Accurate quantification of the receptor amount and fiber density was investigated in a pilocarpine model of TLE in mice, where both the density of fibers and the number of CB1 receptors were shown to be increased in the dentate gyrus of sclerotic hippocampi, but only in inhibitory terminals establishing symmetric synapses [42].

**Figure 5:**
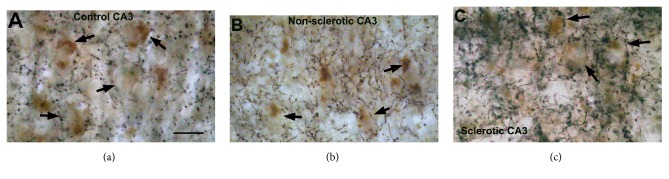
Light micrographs show the distribution of CB1-immunopositive fibers in the human control (a) and epileptic (b-c) CA3 regions. (a) Homogeneous CB1-immunopositive meshwork was present around the pyramidal cells. (b) In epileptic patients without hippocampal sclerosis the density of CB1-positive fibers has been increased moderately. (c) Further increase in density has been observed in the CA3 region of the sclerotic hippocampi, if pyramidal cells were preserved. Arrows point to pyramidal cell bodies surrounded by basket-like formations of CB1-positive fibers. Scale bar: 20 *μ*m.

**Figure 6:**
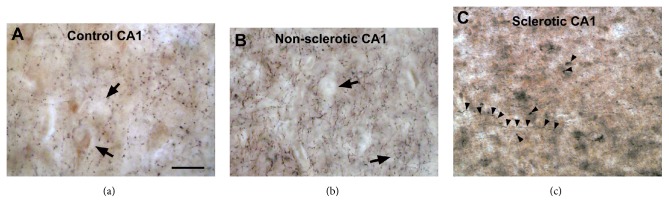
Light micrographs show the distribution of CB1-immunopositive fibers in the human control (a) and epileptic (b-c) CA1 regions. (a) CB1-immunopositive axonal network was present in the stratum pyramidale. (b) In epileptic patients without hippocampal sclerosis the density of CB1-positive fibers has been slightly increased. (c) The density of CB1-positive axons has been decreased in the sclerotic CA1 region. Arrows point to pyramidal cell bodies surrounded by basket-like formations of CB1-positive fibers. Arrowheads show axons. Scale bar: 25 *μ*m.

Changes of the CB1 receptor-expressing fibers of other regions of the human hippocampus were not examined quantitatively in epilepsy. The qualitative light microscopic observation however showed similar changes in the CA3 region of both sclerotic and nonsclerotic hippocampi (Figure 5) to those we found in the dentate gyrus. The homogenous fiber network has been enhanced and became inhomogeneous in the stratum pyramidale and formed extremely strong meshwork around individual cells (Figure 5(c)). Similar extensive networks were found around individual mossy cells in the hilus of the dentate gyrus (Figure 4(d)). The nonsclerotic CA1 region displayed similar density and distribution of CB1-positive fibers (Figure 6); however, in Type 2 patchy samples inhomogeneous fiber density was also observed. The sclerotic CA1 region still contained scattered CB1-immunopositive fibers (Figure 6(c)). In the lack of electron microscopic examination, the synaptic targets and function of these CB1 fiber networks have not been determined.

## 3. Conclusion

Our results show that both PV-containing and CB1-expressing perisomatic inhibitory cells are preserved in the epileptic hippocampi.

Although the number of PV-positive cells has been decreased in parallel with the degree of sclerosis, their axon terminals were present and even sprouted in the dentate gyrus, innervating larger numbers of granule cell somata and axon initial segments, than in the control. The presence of hypercomplex basket and chandelier axonal formations in the epileptic hippocampus also supports the sprouting of perisomatic axons [15, 31]. In the CA1 and CA2 regions PV-immunoreactive cells and their axons survive as long as their postsynaptic targets, the pyramidal cells, are present; they are missing exclusively in the sclerotic CA1 region. Somatic and axonal inhibitory input also remained unchanged (or slightly sprouted) in the epileptic CA1 and CA2 regions with surviving pyramidal cells. However, regional differences can be seen in the ratio of PV-positive axon terminals contacting principal cells in the hippocampus: 23 to 45% of the inhibitory boutons are PV-stained in the CA1 region, whereas only 18% in the CA2 region and 11 to 12% are PV-stained in the dentate gyrus.

The decrease in PV-positive cell numbers has been shown in animal models of epilepsy [33–35, 43]. The calcium overload of the cells—resulting in conformal changes of the parvalbumin molecule—was supposed to be the cause of reduced immunoreactivity in animals [34, 35]. Our electron microscopic results indicate that similar mechanisms might operate in the human epileptic hippocampus; that is, the reduction of PV-positive elements in epilepsy is likely due to lack of PV-immunostaining rather than to interneuronal cell death. CB1-R-expressing fibers were also shown to be sprouted in all subfields of the epileptic hippocampus, except the sclerotic CA1 region. However, the CB1-R-immunostained cells and fibers are present even in this region of the sclerotic patients, showing remarkable preservation of this cholecystokinin-containing perisomatic inhibitory cell type.

Although inhibitory input of principal cells was preserved in the human epileptic hippocampus [17–19], this does not imply that perisomatic inhibition remained unchanged. Changes in GABA-A receptor subunits in the human epileptic hippocampus [44, 45] suggested perturbations in GABAergic signaling at the subcellular levels, with possible functional consequences for perisomatic inhibition. Electrophysiological recordings in the human epileptic hippocampal formation confirmed that inhibition is altered in human epilepsy. Impaired inhibition was found in the dentate gyrus in parallel with mossy fiber sprouting [46, 47], and weak or absent inhibition was found in the CA2 region in one study [48], whereas functional but modified inhibition was found in another paper [19]. Furthermore, depolarizing and hyperpolarizing inhibitory synaptic potentials were found in the subiculum [49, 50], contributing to the generation of interictal activity in vitro.

Different types of interneurons show different vulnerability to epileptic injury. Calbindin-positive dendritic inhibitory cells are considered as one of the most resistant cell types in epilepsy [24, 32, 39]. Numerous calbindin-stained interneurons were found throughout the epileptic hippocampus, including the sclerotic CA1 region as well (lacking pyramidal cells), and were shown to change their postsynaptic targets from principal cells to surviving interneurons, including themselves [32]. The considerable sensitivity of the somatostatin and neuropeptide Y-immunostained dendritic inhibitory cells was the first description of the selective vulnerability of specific interneuron types [51–53]. These neurons are mainly located in the dentate hilus and the stratum oriens of the Cornu Ammonis and almost exclusively overlap with HIPP and O-LM cells [1]. Later, the calretinin-containing interneuron-specific inhibitory cell type was also shown to be very sensitive in human temporal lobe epilepsy [54]. Regarding our results, we can conclude that perisomatic inhibitory cells are less vulnerable to epilepsy, particularly in the dentate gyrus, where both basket and chandelier cell axons were demonstrated to be considerably sprouted. However, we have to note that PV-positive interneurons are heterogeneous in point of view of vulnerability to epileptic injury. Neurons located in the hilus and in the stratum oriens of the CA1 region (note the same location as somatostatin-positive interneurons) were found to disappear from the nonsclerotic epileptic hippocampus as well (Types 1 and 2). Although the overall density of PV-immunopositive cells decreased in the epileptic hippocampus [17, 18, 25], the perisomatic inhibitory input analysis of the principal cells at electron microscopic level suggested that these cells survived as long as their postsynaptic targets, that is, the principal cells, are present, but they lost their PV-immunopositivity [17–19]. The preservation of perisomatic inhibitory cells might be a compensatory mechanism in epilepsy, to increase the control of firing activity of principal cells [12]. However, the increased amount of perisomatic inhibitory input together with functional modifications of GABAergic inhibitory processes may explain that TLE patients are frequently therapy resistant [55]. The most often applied antiepileptic drugs target the GABAergic system and try to enhance it [11]. Perisomatic inhibitory input has been already enhanced in these patients and further enhancement of the GABAergic inhibition rather increases the possibility of epileptic discharges than decreases it, since perisomatic inhibition is responsible for the synchronous firing of the cells [3].

## Figures and Tables

**Table 1:** Distribution of the target elements of PV-positive terminals (%). Changes in the target elements of PV-positive terminals were determined in human control and epileptic dentate gyrus and CA1 region by examining 42 to 107 boutons per patient per region. Percentages were formed for each control subject and epileptic patient and were then averaged. Note that relatively high ratio of PV-positive boutons terminated on other cellular compartments compared to the perisomatic region (i.e., large numbers of terminals gave synapses to dendrites and spines).

		Number of boutons examined (number of subjects or patients)	Soma (%)	Dendrite (%)	Axon initial segment (%)	Spine (%)
DG	Control	105 (2)	32.6 ± 3.0	44.3 ± 4.9	15.9 ± 5.2	7.2 ± 7.1
	Epileptic	213 (4)	14.5 ± 9.0	45.4 ± 8.8	31.3 ± 9.9	8.8 ± 5.8

CA1 region	Control	215 (4)	14.8 ± 6.9	53.1 ± 6.2	14.5 ± 8.0	17.6 ± 8.0
	Epileptic	275 (4)	11.1 ± 4.6	60.8 ± 7.5	17.3 ± 9.6	10.9 ± 6.7

**Table 2:** Synaptic input of hippocampal principal cell somata and axon initial segments. The synaptic coverage (synaptic length/100 *μ*m soma or AIS perimeter) was calculated for each cell body or AIS as follows. All cell bodies or AISs found in one electron microscopic section (with an area of about 300 × 300 *μ*m) were photographed. The perimeter of these profiles, as well as the synaptic length of all boutons contacting them, was measured offline, with the aid of the ImageJ program (NIH). The sum of symmetrical and asymmetrical synaptic lengths per 100 *μ*m soma or AIS perimeter gave the inhibitory and excitatory synaptic coverage, respectively. Data deriving from different patients but falling within the same patient group (control, Types 1–3) were pooled and averaged. N/A = data not available.

	DG					CA1				CA2		
	Soma inhibitory synaptic coverage (*n* = granule cell somata examined)	PV%	Soma excitatory synaptic coverage	AIS inhibitory synaptic coverage (*n* = AIS examined)	PV%	Soma inhibitory synaptic coverage (*n* = pyramidal cell somata examined)	PV%	AIS inhibitory synaptic coverage (*n* = AIS examined)	PV%	Soma inhibitory synaptic coverage (*n* = pyramidal cell somata examined)	PV%	Soma excitatory synaptic coverage
Control	0.53 ± 0.6 (50)	11.3	0	0.47 ± 1.68 (183)	12.2	0.64 ± 0.47 (120)	23.1	2.34 ± 2.55 (177)	45.4	0.80 ± 0.41 (28)	17.5	0
Epileptic (T1-mild)	0.67 ± 0.75 (32)	8.1	0	0.92 ± 1.96 (74)	10.5	0.64 ± 0.52 (119)	33.2	3.14 ± 3.64 (157)	42.5	N/A	N/A	N/A
Epileptic (T2-patchy)	0.67 ± 0.61 (31)	2.7	0	1.29 ± 2.39 (58)	12.5	0.42 ± 0.40 (79)	46.6	2.59 ± 3.51 (102)	38.9	0.81 ± 0.55 (21)	6.3	0.46 ± 0.68
Epileptic (T3-sclerotic)	0.71 ± 0.70 (33)	4.0	0.05 ± 0.14	2.60 ± 4.11 (144)	25.1	N/A	N/A	N/A	N/A	0.72 ± 0.51 (28)	0	0.31 ± 0.48
